# Supplementary material for: Choosing fast and simply: Construction of preferences by starlings through parallel option valuation
Source: PLoS Biol. 2020 Aug 24;18(8):e3000841. doi: 10.1371/journal.pbio.3000841 (PMC7480835; doi:10.1371/journal.pbio.3000841)
Supplement: S1 Text — Computational description, analytical implementation and numerical simulations. SCM, sequential choice model. (PDF) [file pbio.3000841.s001.pdf]

Supplementary text 1

The Sequential Choice Model (SCM)

1. Brief description

The Sequential Choice Model (SCM) postulates that decision-makers make no comparative evaluation of the options at the time of choice. Instead, the same mechanism operating when only one option is available is deployed in parallel for each option present. Much in the same tradition as classical optimal foraging models, the SCM assumes that the main selective pressure modelling decision mechanisms is the resulting rate of net objective gains. In turn, a maximal rate can be achieved by treating each encounter with a potential prey as a choice between engaging with that opportunity and letting it pass to pursue other options that the environment may offer in the future. The critical variables are thus the ratio of gain to engagement time typical of a class of prey (called its "profitability") and the ratio of expected gains to expected time in the environment as a whole (the Long Term Rate *(1)*). This however is a mathematical description of a theoretical rate-maximizing policy, not an explicit hypothetical model of behaviour mechanisms.

In the hypothetical mechanism underlying the SCM, the agent develops a subjective valuation of each option across sequential encounters (modelled as single-option trials in our paradigm) through learning. This valuation is sensitive to the option's absolute properties (i.e., more profitable options are valued more highly; *(2-4)*), to the animal's energetic state during learning (i.e., options found under greater need are valued more highly; *(5-8)*), and to the memory of other alternatives available in the environment: The subjective value of an option varies inversely with the average gains in the environment; *(9-11)*). Overall, the agent ends up with a library of subjective values for each option. When learning reaches stability, the average time to respond when one option is encountered on its own reflects the option's subjective value or attractiveness, with better options eliciting shorter response times.

When two or more options are met simultaneously (in our protocol this happens in choice trials), the SCM proposes that no explicit deliberation between the options takes place. Instead, each option triggers an independent, non-interfering sequential process (as in single-option trials), and the option generating the shorter response time is expressed behaviourally as a choice. Since there is no observable outcome of the process for the option eliciting a longer RT, the faster response “censors” the original distribution of RTs in the ‘losing’ alternative, to generate an observed distribution where the right tail has been suppressed.

This argument can be formalized somewhat. For simplicity, let us assume that two options, say A and B, are encountered simultaneously and that A is objectively better than B (i.e., has higher profitability). The distributions of response time built during single-option trials will partly overlap, but A will have lower central value. Consequently, when the two sequential processes run in parallel (which is isomorphic to sampling each distribution once), A will ‘win’ more often than B. The distribution of times to choose either option will shift to the left relative to single-option trials, for the reason explained above. That is, the observed time to respond to an option in choice trials should, in general, be shorter than the time to respond to the same option in single-option trials. Since the distribution of RTs for A is shifted to the left, A will be chosen most of the choice trials, and thus will be less censored than B. This means that the leftwards shift in RTs in choice trials respect to sequential trials will be sharper for B than for A.

In summary, the time to accept an option in choice trials will be, on average, shorter than in single-option trials because the right tails of each distribution will be cross-censored in choice trials. This shortening of response times should be particularly noteworthy for the non-preferred option B, because longer times to respond to B are censored more often (for further details see *(12)*). This prediction of the SCM goes against the intuition predicated by all models that assume that a cognitive comparison is effected between the alternatives when a choice is faced. These ideas and predictions are expressed mathematically in the next section.

2. Mathematical (analytical) implementation

Say that two options A and B elicit, when encountered alone, response times well described by the probability density functions f_A and f_B, respectively.

When these two options are met simultaneously, the probability of choosing A is given by the probability, P_A, of a sample from f_A being shorter than one from f_B. This corresponds to

$$P_A = p(l_A < l_B) = \int_0^\infty f_A(t) \cdot [1 - F_B(t)] dt \quad (1)$$

where l_A and l_B are random samples from the respective distributions, F_B is the cumulative probability of drawing a latency larger than t from f_B, and t is a particular response time value.

From *Equation 1* we can predict the distribution of response time for each option in choice trials. Focusing on A and restricting our analysis to binary choices, this is given by the conditional probability that $l_A = t$ given that $l_A < l_B$ in that trial, for all t. Defining R as "$t < l_A \leq t + dt$" and S as "$l_A < l_B$", we seek g_A such that

$$g_A = \frac{h_A(R, S)}{j_A(S)} \quad (2)$$

where h_A(R,S) is the probability of joint occurrence of R and S, and j_A(S) is the unconditional probability of $l_A < l_B$.

Since j_A(S) is given by *Equation 1* and h_A(R|S) can be expressed as

$$h_A(R, S) = f_A(t) \cdot P[l_B > t] = f_A(t) \cdot [1 - F_B(t)] \quad (3)$$

we can write *Equation 2* as

$$g_A = \frac{f_A(t)\cdot[1-F_B(t)]}{\int_0^\infty f_A(t)\cdot[1-F_B(t)]dt} \qquad (4)$$

Equation 4 predicts the distribution of times to respond to A in choice trials with A and B, using their response-time distributions in forced trials. The mean response times in choice are obtained by integration of this function. The same method can be used to find P_B, the probability of choosing B, and its response-time distribution in choice trials, g_B.

Next, we illustrate this mathematical framework using both *Gaussian* and *ex-Gaussian* theoretical distributions. We then resort to a Monte-Carlo procedure to generate the response-time distributions in choice trials.

3. Numerical simulations

We generated two types of theoretical distributions, *Gaussian* (**Figure A1a**) and *ex-Gaussian* (**Figure A1b**). We are aware that the distributions of response times are usually heavy-tailed and thus not well captured by symmetrical distributions as the Gaussian. Yet, we include it only for illustration purposes. The *ex-Gaussian*, on the other hand, is frequently used to model response time (13). For each distribution family, we defined 3 probability density distributions of putative response times for 3 options A, B and C, with profitability of A > B > C. ***Table 1*** shows the parameters used. **Figure A1a** and **1b** show the density functions.

Table 1. Parameters used to generate the probability density functions, f, of response times in single-option trials.

	Gaussian		ex-Gaussian		
	μ	σ	μ	σ	τ
$f_{(A)}$	600	$\mu_{f(A)}$ /5	350	$\mu_{f(A)}$ /10	250
$f_{(B)}$	$\mu_{f(A)}$ * 1.35	$\sigma_{f(A)}$ * 1.35	$\mu_{f(A)}$ * 2	$\sigma_{f(A)}$ * 2	$\tau_{f(A)}$ * 1.25
$f_{(C)}$	$\mu_{f(A)}$ * *1.7*	$\sigma_{f(A)}$ * 1.7	$\mu_{f(A)}$ * 3	$\sigma_{f(A)}$ * 3	$\tau_{f(A)}$ * 1.5
**mean** (f)	μ		$\mu + \tau$		
**standard deviation** (f)	σ		$\sqrt{(\sigma^2 + \tau^2)}$		

Using a Monte-Carlo procedure, we then generated 10^5 putative choices between A and B, A and C, and B and C, respectively. On every iteration, two response times, one per option, were drawn randomly from the corresponding theoretical distributions, $f_{(A)}$, $f_{(B)}$ and $f_{(C)}$ (this procedure was repeated for Gaussian, **Figure A1a,** and ex-Gaussian distributions, **Figure A1b**). The shorter response time was expressed as a 'choice' and its value stored as the corresponding response time (i.e., there is a 'cross-censorship' of the alternative option). Using this method, we built response time probability density distributions, g, for choosing each option in all pairwise arrangements (**Figure A1c** and **A1d**). For visualization, we show only response-time distributions for choosing A in A vs. B (g_A), B in A vs. B (g_B), and C in A vs. C choices (g_C). The simulations yielded higher proportion of choices for the better option in each pair (see top insets in **Figure A1c** and **d**). Importantly, there is a leftwards shift in all choice response-time distributions (compare top versus bottom panels' dashed vertical lines). To quantify this lateral displacement, bottom insets of **Figure A1c** and **A1d** show the difference between choice and single option mean response times for all comparisons (i.e., *delta response time*). The plots show that the distribution of reaction times for each option are more similar to each other

in choices than in sequential encounters, and that this is caused by a stronger leftward shift in the less preferred of the two options in each case.

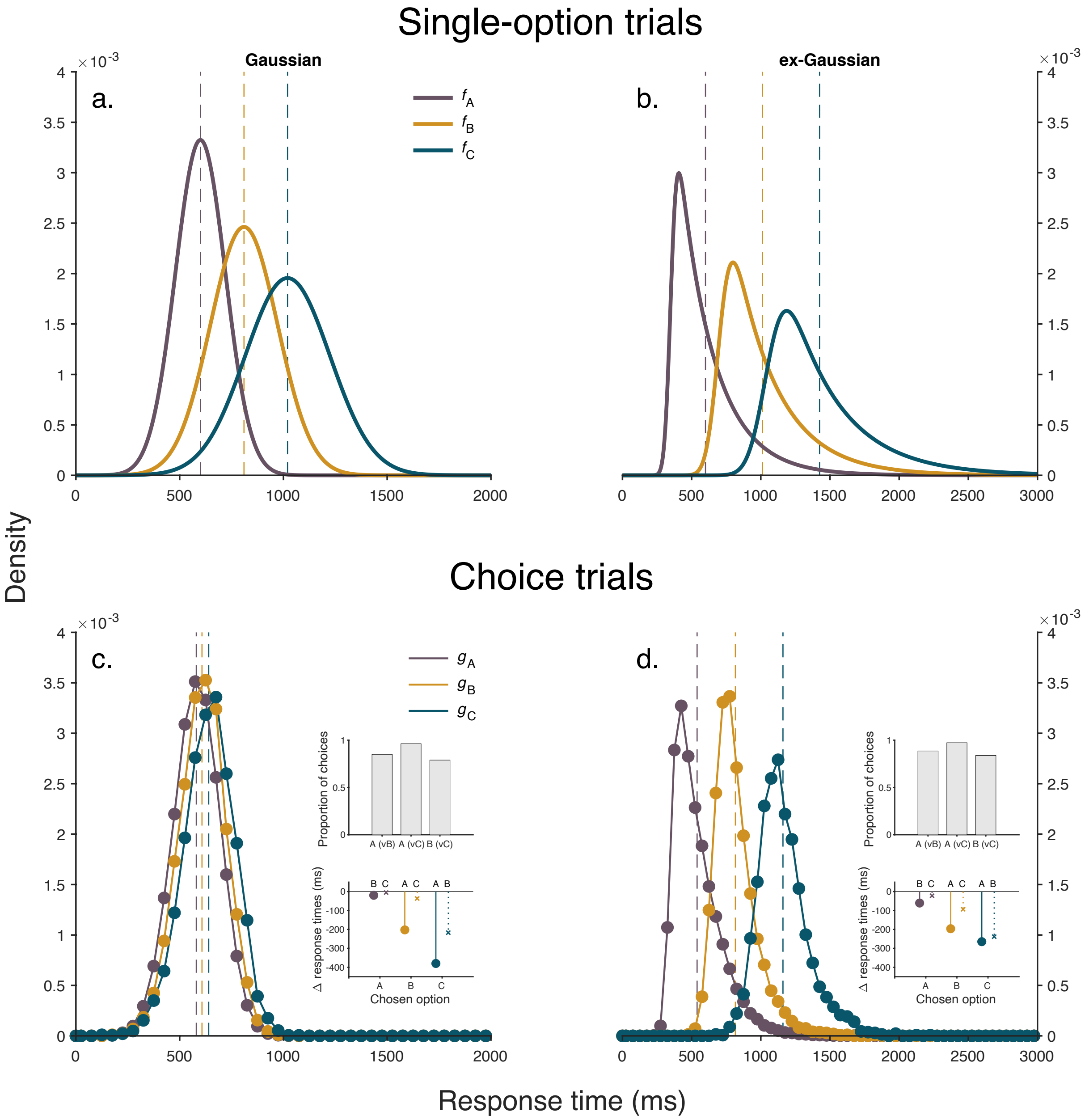

Appendix Figure A1. **Simulated response-time distributions for single-option and choice trials.** **a.** Single-option response time *Gaussian* probability density functions, f_x, with $x = \{A, B, C\}$. **b.** Same as in a. but using *ex-Gaussian* distributions. **c.** Choice response-time probability density functions, g_x, with $x = \{A, B, C\}$, drawn from the corresponding Gaussian distributions depicted in a. g_A corresponds to response times for choosing A in A vs B choices, g_B to choosing B in A vs B, and g_C to choosing

C in A vs C. Top inset shows the average proportion of putative choices for the simulations. Bottom inset shows the difference in mean response times for each option in a pair minus the corresponding mean response time in single-option trials, with dots corresponding to the distributions shown. **d.** Same as in c. for response times drawn from *ex-Gaussian* distributions depicted in b. Dashed vertical lines in each panel show the mean of the matching colour distribution.

References

1. Stephens DW, Krebs JR. Foraging Theory. Princeton University Press; 1986.

2. Bateson M, Kacelnik A. Rate currencies and the foraging starling: the fallacy of the averages revisited. Vol. 7, Behavioral Ecology. 1996. p. 341–52. Available from: http://dx.doi.org/10.1093/beheco/7.3.341

3. Shapiro MS, Siller S, Kacelnik A. Simultaneous and sequential choice as a function of reward delay and magnitude: normative, descriptive and process-based models tested in the European starling (Sturnus vulgaris). J Exp Psychol Anim Behav Process. 2008 Jan;34(1):75–93.

4. Vasconcelos M, Monteiro T, Aw J, Kacelnik A. Choice in multi-alternative environments: A trial-by-trial implementation of the Sequential Choice Model. Vol. 84, Behavioural Processes. 2010. p. 435–9. Available from: http://dx.doi.org/10.1016/j.beproc.2009.11.010

5. Aw J, Vasconcelos M, Kacelnik A. How costs affect preferences: experiments on state dependence, hedonic state and within-trial contrast in starlings. Vol. 81, Animal Behaviour. 2011. p. 1117–28. Available from: http://dx.doi.org/10.1016/j.anbehav.2011.02.015

6. Marsh, B, Schuck-Paim, C., Kacelnik, A. Energetic state during learning affects foraging choices in starlings. Vol. 15, Behavioral Ecology. 2004. p. 396–9. Available from: http://dx.doi.org/10.1093/beheco/arh034

7. Pompilio L, Kacelnik A, Behmer ST. State-dependent learned valuation drives choice in an invertebrate. Science. 2006 Mar 17;311(5767):1613–5.

8. Vasconcelos M, Urcuioli PJ. Deprivation level and choice in pigeons: a test of within-trial contrast. Learn Behav. 2008 Feb;36(1):12–8.

9. Fantino E, Abarca N. Choice, optimal foraging, and the delay-reduction hypothesis. Vol. 8, Behavioral and Brain Sciences. 1985. p. 315–30. Available from: http://dx.doi.org/10.1017/s0140525x00020847

10. Pompilio L, Kacelnik A. Context-dependent utility overrides absolute memory as a determinant of choice. Proc Natl Acad Sci U S A. 2010 Jan 5;107(1):508–12.

11. Vasconcelos M, Monteiro T, Kacelnik A. Context-Dependent Preferences in Starlings: Linking Ecology, Foraging and Choice [Internet]. Vol. 8, PLoS ONE. 2013. p. e64934. Available from: http://dx.doi.org/10.1371/journal.pone.0064934

12. Kacelnik A, Vasconcelos M, Monteiro T, Aw J. Darwin's "tug-of-war" vs. starlings' "horse-racing": how adaptations for sequential encounters drive simultaneous choice [Internet]. Vol. 65, Behavioral Ecology and Sociobiology. 2011. p. 547–58. Available from: http://dx.doi.org/10.1007/s00265-010-1101-2

13. Ratcliff R. Methods for dealing with reaction time outliers. Psychol Bull. 1993 Nov;114(3):510–32.
