# Supplementary figures and images for: Choosing fast and simply: Construction of preferences by starlings through parallel option valuation
Source: PLoS Biol. 2020 Aug 24;18(8):e3000841. doi: 10.1371/journal.pbio.3000841 (PMC7480835; doi:10.1371/journal.pbio.3000841)

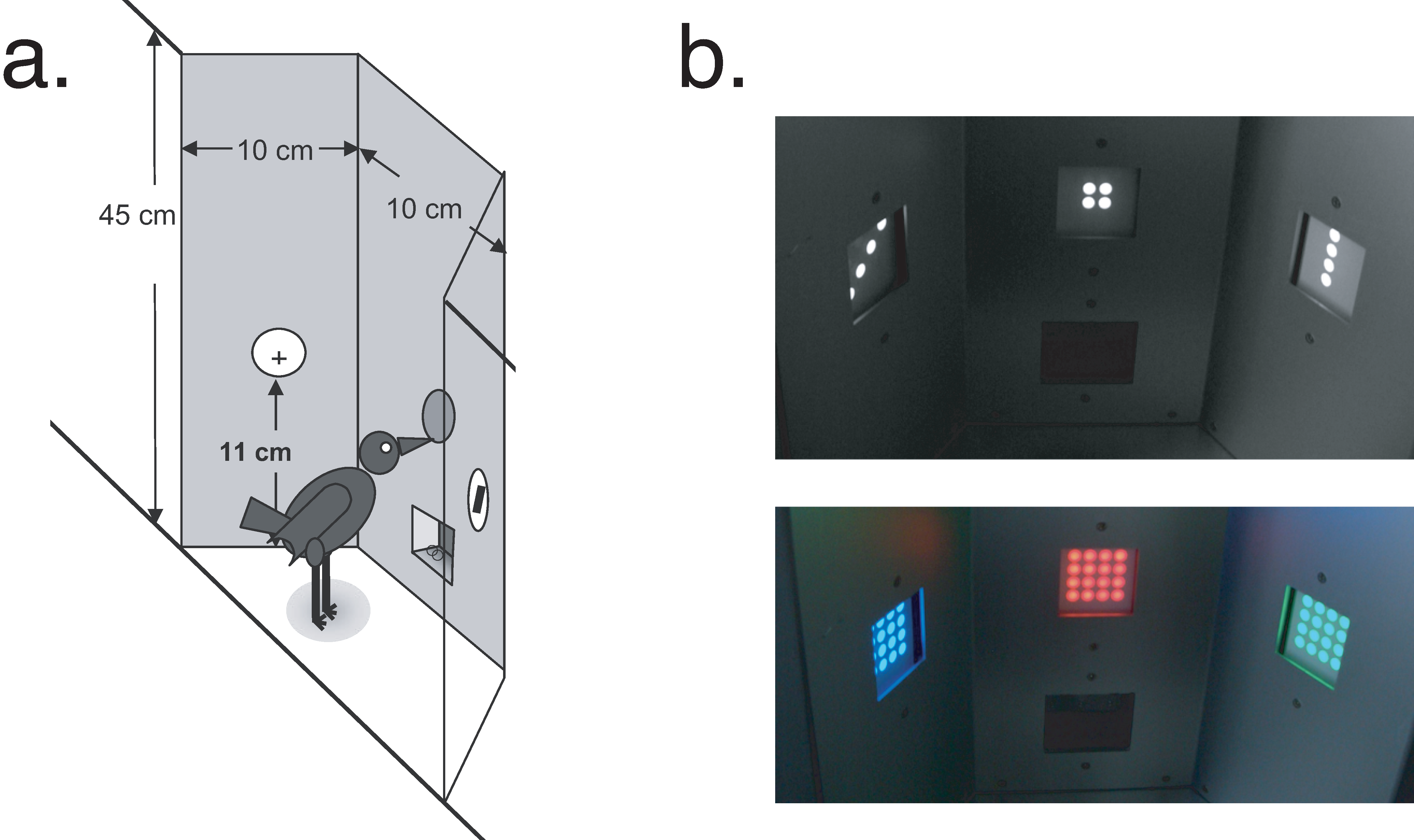

Supplement: S1 Fig — a. Schematics of the operant panels (adapted with permission from [15], APA). b. Close-up photographs of the operant panels displaying different symbols (top) and colors (bottom). (TIF) [file pbio.3000841.s002.tif]
