# Supplementary material for: Choosing fast and simply: Construction of preferences by starlings through parallel option valuation
Source: PLoS Biol. 2020 Aug 24;18(8):e3000841. doi: 10.1371/journal.pbio.3000841 (PMC7480835; doi:10.1371/journal.pbio.3000841)
Supplement: Attachment — Submitted filename: Response to Reviewers.pdf [file pbio.3000841.s004.pdf]

Response to Reviewers (in blue):

<u>Reviewer #1</u>: Monteiro et all provide a very interesting set of results regarding the nature and mechanisms for decision making by using birds as an animal model. *They find that choices are not made on the spot but rather reflect some intrinsic preferences. Additionally, they find that decision making in their task does not involve direct comparison between alternatives*, and rather the behavior can be explained by a model where options are selected randomly one from each other. In general, the paper is well written, although I think that the claims about generality to other conditions could be toned down slightly for a more balanced manuscript.

We do not claim that options are selected randomly, but independently. The reviewer (our italicised section) correctly summarises our two main contributions. We think the results likely to apply to many other systems because the rationale leading to our predictions is not species-specific, but of course data collected in one species cannot prove how general the findings will prove to be. We do not claim that the SCM model must apply to all situations. Our conjecture that it may apply to an important fraction of experiments with humans is presented as such, and justified conceptually, and is of course subject to further testing. We have reworded the final paragraphs to make this clearer.

The description of the SC Model is difficult to follow. It is described in previous literature, but I think that a simplified mathematical description of it in the SI will be valuable for the readers. In addition, one could use that opportunity to develop the basic predictions and their underlying intuitions.

We accept this criticism. We have added a computational description of the model in an Appendix, together with novel, quantitative predictions.

Indeed, *the prediction that RTs should be shorter for pair vs single decisions is a very strong one*. However, I have a number of concerns related to its experimental testing. First, the prediction is qualitative, rather than quantitative. However, the SC model should predict by how much one expects to reduce RT. I think that making a semi-quantitative analysis of the model and the data would be important. Otherwise, it is unclear whether short RT times in pair vs single choices provides really support in favor of the SCM and against the DDM, or whether it provides support for a different interpretation of the results (that I develop below).

As the referee states, the prediction is very strong. This is because the alternative model(s) predict a different sign of the change, namely an increase in RT between sequential and choice encounters while the SCM predicts a decrease. Thus finding a decrease is strong. Quantitative predictions can be implemented but in our view they would not help in this case, for the following reason: As with any quantitative model, predicting how much should RT decrease depends on fitting individual parameters, in sharp contrast with the qualitative prediction, that, predicates that whatever each subject's reaction time distributions, the shift should be towards shorter latencies in choices. The qualitative prediction is clear and very general. We have now

incorporated a semi-quantitative simulation that shows that the likelihood of obtaining the shifts we get under a null hypothesis that choice and single-option latencies come from the same distribution is extremely low. The competing hypothesis does not make quantitative predictions (nobody can tell how long should cognitive deliberation be) other than implicitly expecting the shift to be in the opposite direction to that observed.

I am concerned that the shorter RTs observed in the pair choice condition is due to some attentional effects that do not have anything to do -a priori- with the choice mechanics. In the experimental setup, in pair conditions there is more physical stimulation than in single conditions -there are two choices and the amount of light over the display, if I understand well, is also larger. It can happen that more stimulation can increase attention and therefore reducing RTs. I wonder whether if one can control from arousal/attentional effects by controlling for differences in low level features of the stimulus (such as total brightness of the choices between single and pair conditions), or whether actually the authors have already taken care of this.

The referee agrees that our results contradict the predictions of the ‘construction of preference’ hypothesis, but suggests that they could be generated by an alternative to our proposed model. This is an interesting suggestion but we think that their suggestion does not fit our data. We test reaction times for one stimulus alone versus two stimuli, and as the referee points out adding a stimulus adds stimulation. We cannot control for this. However, if the extra stimulation caused an indiscriminate increase in attention, shortening of reaction times should shorten equally for the majority and minority choice options, while our model predicts that the cross-censorship effect should be stronger for the more rarely chosen alternative, as observed.

I have some comments about Figure 1. I would propose using the same color code in panels b and c. Right now it is quite confusing using colors for one panel, and gray levels for the other.

We are grateful for these comments and have thought carefully about them, but it is not possible to communicate the same information following the referee’s suggestion.

Coloured and gray hues mean different things in the figure, with gray shades corresponding to different profitability (colour code maintained for panels d. and f.), and coloured rectangles in panel b. correspond to the time delay associated with each alternative (and are maintained in panel c, where we show response rates as a function of each option’s associated delay). We did, however, replace the gray shades in panel e, so that each hue is exclusively used with a single type of data.

In panel e, it is difficult to see the large differences in RT. I think it would be better to plot RT for single vs RT for pairs, and see that this line has lower slope than one, or something on those lines.

Again, we thought carefully about this suggestion, and as you can see below, we tried a number of alternatives including the plot suggested by the referee (leftmost panel). Not surprising, replotting the same data in different formats shows the same results, but each plot has its advantages and disadvantages. The figure we included in our submission (Figure 1 Panel e) is the only format that presents both individual and group data, that shows effects for each of the 6 option types, and that allows a visual general impression of the overall result. We used a ‘heat’ figure, that may be more complex to read precisely because it contains more information, but all the information conveyed is important, and we prefer to stay with that format.

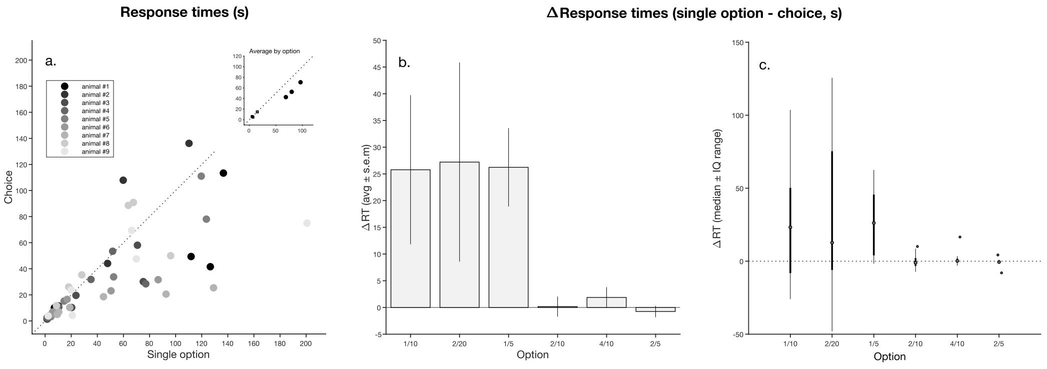

<u>Minor comments</u>:
-I don't see how reference 6 first into supporting Systems 1 vs Systems 2 hypothesis.

Thank you, this was an error and it is now corrected.

Also I would say that this distinction is largely controversial, at least many people think that System 1 is not sloppy and rather follows the rule of automatic Bayesian inference.

We agree that how System 1 may work, and even whether there is a sharp real life distinction between two discrete systems is controversial. The controversy is not our topic in this study, but we use the distinction here because it is a very widespread concept, familiar to a large proportion of readers in different fields. We wish to express that our results are relevant to some decisions taken by humans but not to all of them, and Kahneman’s well-known distinction is a quick way to introduce that. The notion of preference construction, which we dispute for our system, may still apply to complex decisions calling for cognitive elaboration. Kahneman’s distinction between the two systems simply helps to make our reasoning transparent.

-In the main text the definition of profitability is not very transparent. I would define the mathematical terms in the text by using symbols, and just spell out the definition of profitability using an equation.

We follow the textbook definition of profitability used in the sources we cite, and we give the definition. We have added wording to better explain why we need to introduce a distinction between ‘profitability ratio’ and plain ‘profitability’. The latter is insensitive to anything other than the ratio of gain to time cost, while the former serves to test the Small-Sooner vs Large-Later crucial to discuss impulsivity, because it depends on the absolute numbers involved and not just their ratio. Our present wording should avoid any potential confusion.

-In the list references 26-28 for reward maximization in sequential decision making, Drugowitsch et al, Journal of Neuroscience, 2012 could be a missing relevant reference.

Thank you, this reference has been added.

Reviewer #2: This paper addresses the interesting topic of whether a non-human animal will show processes similar to the fast and slow decision processes famously reported for humans. As discussed, humans have been found to show failures of procedural invariances in their decision making and have been suggested to make different decisions depending on which system is activated.
In this research, starlings learned six options that differed in amount and delay to food. Latency to peck on single option trials was predictive of preference on binary choice trials -showing procedural invariance - and latencies were shorter on choice trials than when a single option was presented - inconsistent with a deliberation process on choice trials. The birds also did not show an irrational tendency to choose smaller sooner rewards.

The topic is important, the results are clear, and there are interesting messages in the paper, for example that simultaneous decisions are likely rare and less important in nature. However, I have several problems with the current manuscript, some of which likely stem from the short report format that does not provide space to clarify and qualify statements.

For example, "irrational impulsivity" is introduced in the abstract as a human problem. The end of the abstract suggests that irrationality in humans could be due to the use of description. The authors may be just referring to procedural invariance here but since impulsivity is labelled as irrational this is certainly not clear. I think a reader who doesn't know the literature would conclude from reading the paper that impulsivity is a human problem and that these results suggest that since starlings don't show it in an experience-based tasks that it may be because of the use of described problems for humans. What is not discussed is that other animals also show impulsivity and humans do too in experience-based tasks (e.g. Jimura et al., 2009). So I think more needs to be said about how and why the starlings appear to differ from not only humans on described choices but also other animals and humans on experience-based tasks.

We have added an operational definition of irrational impulsivity in the abstract and early in the main text. Briefly, we define it as a preference for immediacy that causes a loss in overall benefit. Others have used the expression “maladaptive impulsivity”

but the qualifier seems to us largely unnecessary and we used both irrational and maladaptive only rhetorically. If a decision trait is not harmful in some way, then it is not impulsivity. Some confusion could have been caused because in the animal literature authors sometimes use impulsivity narrowly for the task in hand, without reference to an ecologically justified optimality criterion such as rate of gains per unit of time. An extensive discussion is not pertinent here, but by using a clear definition that is rooted in foraging theory, our text should not be controversial. Notice that under our definition discounting reward size by the reciprocal of time costs is not impulsive, but discounting more steeply than required for rate maximization would be.

I am also not clear on what aspect of these results are novel. The main findings stated in the abstract are: "(1) ranking alternatives through direct rating (response time) accurately predicts preference in choice, overcoming failures of procedural invariance; (2) preference is not constructed at choice time nor does it involve time (we show that the opposite is true); and (3) starlings' choices are not irrationally impulsive, but instead directly interpretable in terms of profitability ranking ". Certainly, the first two of these have been shown and argued in some of the authors' previous work. Thus I think that the one-sentence conclusion: "The hypothesis that preferences are built at choice time is contradicted by data on starlings' decisions by experience" has been reached previously. If not, then it needs to be explained how this goes beyond what was done before to reach this conclusion. For the third finding I would like to know more about how it differs from Shapiro et al., 2008.

Our study is novel procedurally, conceptually, and in its results. Procedurally, this is the first study where multiple option types share an environment (in Shapiro et al's tests animals were trained and tested with only two options). The present configuration emulates the natural foraging scenario assumed in foraging theory models, while Shapiro et al's protocol followed the tradition of psychological experiments that focused on binary choice. Conceptually, the formal presentation of the SCM in the Appendix is novel and more precise than anything discussed before. Finally, at least for this family of protocols, our results settle the superiority of the SCM respect to models that predicate a comparative evaluation of the options with construction of preference at choice time.

I suspect that these concerns are because the authors did not have the space to properly discuss the research novelty and implications but I don't think it should be published until it is better explained.

We agree, and appreciate the comment. Within the space limits, we have mitigated this deficit by adding an explicit theoretical appendix and references to it in the main text.

Reviewer #3: This paper presents an analysis of choice and response time in Starlings under different delays and magnitudes. I think these data are valuable, but the paper is confusingly written, making it difficult for me to assess its contribution.

My impression is that the authors are making very strong claims about the nature of decision making beyond what the data support.

This impression is at variance with the perception of the other referees. We are assertive about our results for the experimental conditions, and offer speculative conjectures about their significance elsewhere, making that clear.

Major comments:

The exposition could use some work. A number of concepts are introduced in the first paragraph (procedural invariance, decisions from description vs experience, willingness to pay) without explanation. I think the authors should introduce these concepts more systematically.

Because this paper touches on several research fields, we introduce the concepts briefly and give references to our sources. These concepts are widespread in decision research and we define each of them as well as pointing to references. It seems unnecessary to introduce a lexicon table, but we could add a terminological appendix if the editor would favour it.

p. 3: "A consequence of these assumptions is that response times towards a given stimulus when picked out of a choice should be shorter than when the same stimulus is encountered alone." I must confess that, based on the description of the model in the preceding paragraph, I don't see where this prediction comes from.

This difficulty should be solved by our new Appendix.

p. 3: what is "cross-censorship"?

This should be clear from the Appendix.

p. 4: "In the pairwise comparisons where the profitability of the options was equated, the birds in fact showed the opposite: they preferred the larger-later alternative over the smaller-sooner one. This result contradicts prevalent ideas on impulsivity but supports reward rate maximization with partial account of the common time intervals." I see a few problems with this statement. First, a preference for larger-later does not mean that the animals not impulsive (they could still have a time preference expressed as a discount function), although it's not clear from this statement what exactly the alternative account is or what it predicts. Second, the reward rate maximization account is not explained enough to understand what it does or doesn't predict relative to other models. If one is going to discuss computational models in a paper, it would be very useful to actually simulate the models and fit them to data, compare the data to other models, and so on.

The referee seems to imply that to "have a time preference expressed as a discount function" qualifies as impulsivity. This does not fit our definition of impulsivity, which is narrower. Choice based on rate maximization is often considered optimal behaviour and does require discounting rewards by the time it takes to process

them. The very notion of profitability in optimal foraging theory is a time-discounting criterion. We have made this clearer in the text. The SCM is now described in full computational detail in the Appendix. The critical predictions of SCM do not require a simulation as they are theoretically derived from the model's assumptions. We have introduced a simulation procedure to support our statistical analysis.

Showing procedural invariance using a response time measure does not necessarily contradict the results with humans where choice is the dependent measure. In any case, I think the onus is on the authors to demonstrate why you get procedural invariance in one situation/species and not another. This has to be more than just hand-waving about System 1 vs. System 2.

We feel that this criticism and reference to hand waving are unfair. We do not present novel data on humans, just as the canonical papers predicating the construction of preference in humans do not report novel data on birds. But we do relate the results conceptually. It would of course be impossible to replicate experiments by description in starlings. We present experiments with a different animal species in which the results reported in humans have an opportunity to present themselves, but they do not. We present a conjecture (identified as such) about potential results of human experiments that used protocols similar to those used in our animals. It seems to us that the onus is on scholars working on humans to follow our conjecture and try it out. If they did replicate the results we get in birds, that would be impactful in interpreting how the human results come about. The reference to the two systems aims at being clear that even our conjecture is limited: we acknowledge that some human decision-making obviously does not fit our model, for instance, the kind of decisions widely labelled in the human literature as System 2. Why is this hand-waving?

I found it curious that the authors contrast their work with "human experiments, which are mostly based on description rather than experience" (p. 5). But they neglect to mention that there is a now enormous human experimental literature on the description-experience gap. I see only one paper from this literature (Ludvig & Spetch, 2011) cited here. The influential review by Hertwig & Erev (2009) has already been cited over 600 times.

We don't refer in the quotation to all human experiments, but to those related to the issue at hand, that of construction of preference. As we wrote, "most" of such work, including the seminal works of Tversky, Kahneman and Slovic give people choices in one-shot lotteries. Slovic's crucial paper on the construction of preferences and the problem with procedural invariance is based on such methods. This has been questioned by authors such as Hertwig and Erev (which we should have cited, and now do), but our arguments introducing the critical comparison between reaction times in choice and non-choice tests have not been discussed in this respect.

I think it is problematic to call response time a "rating". This is meant to draw an analogy to human desirability ratings, but it's not clear to me that these are measuring the same thing.

This does not seem problematic to us. Reaction times (RT) are not by themselves a rating. Options can be rated in order of reaction times, and this is what we do. This is also what Slovic and others do when ranking options according to willingness to pay (WTP). WTP is a metric used for direct rating, and RT is another metric also used, by us, for direct rating. Direct rating means rating an option by how the subject responds to it, and not through a choice. We compare the ranking of options obtained through RTs with the ranking obtained through choice. The two rankings are negatively correlated. We can never be sure whether two behavioural metrics 'measure the same thing' when the thing being measured is labelled 'desirability'. Self-reported desirability is not an option in animal work, and the point of our work is to contrast it with results in choice protocols.

The finding that response time is predictive of choice is not new, at least in the human choice literature. Indeed, this is one of the key phenomena motivating sequential sampling models of choice (some of which the authors cite).

Our study examines a compound of several predictions of SCM: that RT's predict preferences, that RT's in choice are shorter rather than in sequential tests (rather than longer as expected from construction of preferences), that RT parallels willingness to pay in that it can be measured in the absence of a choice, but in contrast with verbally expressed WTP it does not lead to breaches of procedural invariance. And, at a more general level, it is novel that research originating in behavioural ecology establishes both theoretical and empirical bridges to research in human decision science. None of the existing sequential sampling models of choices does this. We are not clear on what the referee is requesting from us here.

There is only one statistical analysis reported in the paper, in the caption of Figure 1, and the results are quite weak: only one comparison yields a p-value below 0.05. This does not lend strong support to the authors' claims.

We now have examined the strength of our claims in a multiplicity of attacks:

> Fig1d: The dashed line shows a Deming regression. We now specify the equation for the estimated model and report the 95% confidence interval for the regression's slope (that does not include a slope of 0).
>
> Fig1e: We have added new bootstrap and sensitivity analyses to support the strength of our result. The analyses are described under Analyses in the Methods section, and we've also added a supplementary figure (Supplementary Figure 3) to report their outcome.

Fig1f: Notice that our focus is to contrast impulsivity, that predicts the three bars to be below 50%, against gain rate, that predicts the opposite. All bars are above 50%. we compared our results against 50%, not against the predictions of impulsivity, because the latter does not make quantitative predictions we can use.

Minor comments:

- p. 2: "maybe endemic" -> "may be endemic".

  Thank you, this has been corrected.

- Sometimes "procedure invariance" is used instead of "procedural invariance"

In Slovic's seminal paper on the construction of preference he writes:

> *'These "preference reversals" violate the principle of procedure invariance fundamental to theories of rational choice and raise difficult questions about the nature of human values. If different elicitation procedures produce different orderings of options, how can preferences be defined and in what sense do they exist? Describing and explaining such failures of invariance will require choice models of far greater complexity than the traditional models.'*

Following this, we now use procedure and not procedural everywhere through the paper.

- Caption of Fig 1: I find it misleading to label p<0.1 with an asterisk.

We agree with the referee, but in fact there was an error in the panel that is now corrected. To clarify, we performed 2-tailed t-tests against 50%, with * = <0.05 and ** = <0.01.

<u>Reviewer #4</u>: This interesting paper reports the results of a food choice experiment in Starlings. The starting point of the paper is the question whether preferences are constructed on the spot during decision making or whether they represent exist some basic approach tendencies that also guide binary choices. If the former is true, then choices should take extra time to perform compared to single-item approach decisions. However, the experiments show that this is not the case. Binary choices can be well predicted using the response times of single-item approach decisions and choices in general reflect profitability rankings of the options. The authors conclude that "irrationalities that prevail in research with humans may not show in decisions by experience protocols».

There is a lot to like about this paper. It formally tests decision theories developed for human choice in an animal population that is not routinely tested and comes up with clean, somewhat surprising results. The methods are all fine and the results are definitively thought-provoking and should be published in some form. However, I do

not fully agree with the framing and interpretation of the results and think the authors would need to change the relevant parts of the manuscript so that this is not misleading.

1) The authors claim their results show that decisions differ between experience-based protocols and protocols by description, and that human research would need to test experience-based protocols to establish whether all previous results supporting preference construction really reflect this methodical difference. The implication of this statement is that findings of human research reflect, at least in parts, linguistic decoding of option descriptions. However, this is misleading. There are numerous experience-based protocols in human research by now, for instance experiments where participants pick between depicted food items and get to actually eat one of them. I cannot see how these protocols reflect language processing any more than the disk-pecking protocol applied here. The authors would need to take this into account when comparing experiments conducted in humans and animals.

We claimed that the critical human data showing failure to procedure invariance, and supporting the hypothesis of preference construction at choice time, are based on experiments by description. We make no claims about other areas of decision research. Indeed, many problems have been explored by both description and experience, and results are mixed: in some cases the results differ and in other they are consistent. Hertwig and Erev's (2009) review states the following: *"It is well-established that not all properties of human behaviour inferred through description-based protocols are confirmed when similar problems are studied by experience.*" We have searched but failed to find references to experiments by experience demonstrating the failure of procedure invariance. We make no claims about linguistic decoding, and we shouldn't, as we collected data on birds. We focus on failures of procedure invariance because, should that be found in our protocol, it would falsify our model of choice. We find that that the failures do not occur, and report additional data supporting our model of choice. We try to be extremely cautious while advancing some conjectures that we present as such.

2) The authors give very little space to discussion of other possible explanations for their findings and their divergence to studies in humans. They do mention possible interspecies differences at least briefly, but they do not discuss whether their findings may be specific to pecking for food. It seems a bit of a stretch to me to draw conclusions based on the pecking protocol about e.g. human decisions where to go on holiday. The manuscript would benefit if the authors could be more precise which types of choices they talk about and which ones are probably fundamentally different and thus not affected by the present findings.

We totally agree that many important aspects of human behaviour, especially those in which obvious deliberation plays a role, such as where to go on holiday or when to stop the coronavirus lockdown, will not be satisfactorily handled by a model of parallel competition and RT cross-censorship. We don't make such claims and tried to make it more explicit in the present version. This distinction is why we used Kahneman's distinction for 2 systems, making it clear that our model does not apply

to what is known as System 2, and may apply to some of the decisions assigned to system 1. Given that some predictions of our model have been supported in mammals (Ojeda and Kacelnik, 2018) it is implausible that they could be specific to pecking responses.

3) The authors give very little space to the explanation of their SCM model and its predictions. In particular, the sentence that the reaction time effects result from cross-censoring of choice alternatives will be next to impossible to understand for naïve readers. The authors should at least provide references to papers explaining this model and its implications in detail when describing their predictions.

We have responded to this request extensively by the addition of an Appendix.

4) The authors claim that animals are very unlikely to encounter several prey at the same time. This is obviously not true for animals who hunt animals in herds and need to decide which animal to go for.

We wrote that "if prey are distributed independently, then the chances of a multiple encounter must be lower than that of facing a choice between prey". This remains true. If and when prey are not independently distributed, choice indeed plays a role, but this doesn't mean that choice is the fundamental, let alone only selective force responsible for shaping foraging decisions.

5) The authors interpret their findings in terms of the involvement of system 1 and system 2, and claim that system 2 is language based. Again, this statement is misleading. There is no direct link between system 2 and language, and it is very well possible to ponder visually-presented choice options without involving language.

We agree with the referee, this statement was not sufficiently clear. We have now corrected it.
