# Supplementary material for: Choosing fast and simply: Construction of preferences by starlings through parallel option valuation
Source: PLoS Biol. 2020 Aug 24;18(8):e3000841. doi: 10.1371/journal.pbio.3000841 (PMC7480835; doi:10.1371/journal.pbio.3000841)
Supplement: Attachment — Submitted filename: Cover letter - revision2.docx [file pbio.3000841.s005.docx]

Dear Dr Gasque,

We would like to re-submit a revised version of manuscript PBIOLOGY-D-20-00163.

Please see below a response to all your remarks, indicating the changes made to the manuscript.

Best regards,

Tiago Monteiro (with Marco Vasconcelos and Alex Kacelnik)

1. *Title.* We appreciate your suggestions for alternative titles, but we propose a new one which we believe will attract PLOS Biology general audience while being closer to the work presented:

“*Choosing fast and simply: construction of preferences by starlings through parallel option valuation*”

2. *Figures.* We have restructured all figures by splitting the main figure panels across 4 new main figures. These also incorporate all data from the old supplementary figures. We have added, where relevant and missing, individual data points, from where means and standard errors were calculated. The main paper is now composed of four main figures with one supplementary (showing details of our experimental apparatus). All figure captions refer to the supplementary data file (i.e., “Source Data.xlsx”), that now contains a legend as well. Small adjustments to the text were made to reflect these changes.
3. *Reviewer 4.* We have added a sentence in the introduction to address Reviewer 4’s concern regarding classic race models. We now specify that our model can be construed as an inhibition-free type of race model. That said, we believe that an in-dept discussion of how our model differs from classic race models is out of the scope of the paper as the main message is that, unlike said models, ours predicts shorter (as opposed to longer) response times and assumes that the mechanisms driving choices are co-opted from sequential (no-choice) scenarios – as stated in the manuscript.
4. *Ethical note.* We have added an Ethics statement in the beginning of the Methods section that includes the ethics committee name and license number.
5. *Production requirements.*  All requirements were addressed.
